# *Bartonella* spp. infection in people with Mild Cognitive Impairment: A pilot study

**DOI:** 10.1371/journal.pone.0307060

**Published:** 2024-08-22

**Authors:** Verina Guirguis, Francesca Pupillo, Siena Rodrigues, Nathan Walker, Heidi Roth, Chance E. Liedig, Richardo G. Maggi, Edward B. Breitschwerdt, Flavio Frohlich

**Affiliations:** 1 Department of Psychiatry, University of North Carolina at Chapel Hill, Chapel Hill, NC, United States of America; 2 Carolina Center for Neurostimulation, University of North Carolina at Chapel Hill, Chapel Hill, NC, United States of America; 3 Department of Neurology, University of North Carolina at Chapel Hill, Chapel Hill, NC, United States of America; 4 Intracellular Pathogens Research Laboratory, Center for Comparative Medicine, College of Veterinary Medicine, North Carolina State University, Raleigh, NC, United States of America; University of Montana, UNITED STATES OF AMERICA

## Abstract

Mild Cognitive Impairment (MCI) is a neurological disorder at the transition between normal cognitive decline and dementia. Despite the potential role of neuroinflammation in the pathogenesis of MCI, infectious triggers remain mostly unknown. Infection with *Bartonella* spp., a zoonotic bacterium, has recently been associated with diffuse neurological and psychiatric symptoms. Given the preferential endothelial localization of *Bartonella* spp. and the role of vascular changes in neurocognitive decline, we hypothesized that there is an association between *Bartonella* spp. infection and pathologically accelerated decline in cognitive function in aging. To test this hypothesis, we collected serological and molecular markers of past and present *Bartonella* spp. infection in a sample of older people with and without MCI. Samples were processed in a blinded way to exclude laboratory biases. Contrary to our hypothesis, people with MCI were not more likely than people without MCI to have an active *Bartonella* spp. infection as measured by droplet digital PCR (*p* = 0.735) and quantitative PCR (*p* = 1). In addition, there was no significant difference in positive serological results between cases and controls (*p* = 0.461). Overall, higher-than-expected active *Bartonella* spp. infection (37% by ddPCR) and seroreactivity (71% by indirect fluorescent antibody assay) were found in people without MCI. Conclusions require caution, as our study was limited by the small number of cases with MCI. Overall, our results identified a higher than previously recognized rate of exposure and infection with *Bartonella spp*. in this older study population but does not support a specific role for such infection in MCI.

## Introduction

Mild Cognitive Impairment (MCI) is a neurological disorder characterized by a cognitive decline that is greater than expected for a person’s age but does not severely impact daily function. Previous studies have indicated that the prevalence of MCI in participants ages 70–89 without a diagnosis of dementia is 16%, with men having a higher prevalence than women [1]. Notably, patients with MCI have nearly a 40% likelihood of developing dementia [2].

Despite high prevalence rates of MCI in the general population, little is known about the etiology of the disorder. Previous studies found consistent upregulation of several neuroinflammatory markers in MCI, but the specific role of neuroinflammation remains unclear [3, 4]. Sustained or excessive neuroinflammation has been found to play a role in neurodegenerative diseases such as Alzheimer’s disease, yet it remains unknown whether it is a causal factor or an outcome of neurodegeneration [5, 6].

In addition to neuroinflammation linked with progression of neurodegenerative diseases such as Alzheimer’s Disease and Mild Cognitive Impairment, [5] other research has hypothesized that sustained neuroinflammation could be due to various causes such as viral, bacterial, or parasitic infections, an aberrant innate immune system response, and other non-infectious environmental factors [7]. In the context of MCI, herpes simplex virus is one example of an inflammatory pathogen that has been associated with increased incidences of MCI, Alzheimer’s Disease, and dementia and very recent antiviral therapy studies targeting this pathogen have produced promising patient responses [8–11]. The role of neuroinflammation in MCI, however, remains elusive, warranting further exploration of the potential role of other infectious agents, as well as co-infections in the disorder’s etiology.

*Bartonella* spp. comprise a genus of bacteria that have previously been studied to a limited degree for their potential neuroinflammatory role in certain neuropsychiatric disorders. These bacteria are predominantly vector borne, zoonotic pathogens. Domestic cats are a frequent reservoir host for *Bartonella henselae*, *Bartonella clarridgeae* and *Bartonella koehlerae*, with *B*. *henselae* as the infectious agent responsible for cat scratch disease [12]. In addition to cat scratch disease, *Bartonella* spp. have been linked to vascular proliferation, vasculitis, and thrombosis, entities that can be associated with a spectrum of neurological symptoms such as slowed memory processing, memory deficit, as well as mood and personality disorders [13]. Furthermore, several case studies have reported associations of central nervous system infection with. *Bartonella* spp. [14–18] A case-controlled pilot study found that people with schizophrenia/schizoaffective disorder were more likely than healthy controls to have *Bartonella* spp. DNA in their bloodstream [19]. Due to the suggested link between MCI and neuroinflammation, as well as the associations found between *Bartonella* spp. infection and other neuropsychiatric disorders, we investigated the relationship between *Bartonella* spp. exposure and MCI. We hypothesized that past and present *Bartonella* spp. infection would be more common in participants with MCI compared to controls.

## Methods

### Participants

This study, conducted at the University of North Carolina at Chapel Hill from November 30, 2021, until February 1, 2023, and was approved by the UNC-Chapel Hill Institutional Review Board (#21–1614). Participants were recruited from the Raleigh-Durham-Chapel Hill area in North Carolina. Controls consisted of similar age study participants without MCI, who were recruited via social media advertisements, flyers, and the Research for Me website, which lists current research opportunities at UNC-Chapel Hill. Participants with MCI were referred to the study by the team’s board-certified neurologists. Controls met inclusion criteria if they were between the ages of 50 to 80 and not currently experiencing symptoms of or receiving treatment for a neurological or psychiatric disorder. Participants with MCI were included if they were 50–80 years old, had a diagnosis of MCI, and scored below 26 on the Montreal Cognitive Assessment (MoCA) which is the common score threshold for MCI [20]. Exclusion criteria for the MCI cohort included a history of stroke, significant head trauma, bipolar disorder, schizophrenia spectrum disorder, or other psychotic disorders. Also, participants could not have current or past alcohol use disorder, substance use disorder, or current use of opioids or benzodiazepines. Participants from both groups could not be currently taking antibiotics or have a history of blood disorders or past serious adverse reactions to blood draws. Participants confirmed exclusion criteria via self-report.

### Study design

Eligibility was first determined via a verbal phone screening where participants provided their verbal consent and a research assistant confirmed that the participant met inclusion criteria. During the in-person visit, participants provided their written consent and research assistants signed the form confirming the consent process, and they completed a series of questionnaires including demographics, health and animal exposure history, the Epworth Sleepiness Scale, the General Anxiety Disorder 7-item (GAD-7), the Patient Health Questionnaire 9-item (PHQ-9), and the Quality of Life Enjoyment and Satisfaction Questionnaire Short-Form (Q-LES-Q-SF). The MoCA was conducted by research assistants certified to administer the assessment. A certified phlebotomist then obtained a blood sample of approximately 8 mL, or participants elected to complete the blood draw during a second visit. All participants were compensated for their participation.

### Specimen processing

#### Serological studies

As described previously, [21, 22] each participant was tested using five indirect fluorescent antibody (IFA) assays, each representing a unique *Bartonella* species or genotype. *Bartonella vinsonii* subsp. *berkhoffii* (genotypes I, and II), *B*. *henselae* (strain San Antonio 2), *B*. *koehlerae*, and *B*. *quintana* IgG antibodies were determined using DH82 cell culture-grown bacteria as antigens and following standard IFA techniques with fluorescein conjugated goat anti-human IgG. A sample was considered *Bartonella* spp. seroreactive at an IFA titer of ≥ 1:64 for any one or more antigen.

#### Molecular studies

Quantitative PCR amplification of the *Bartonella* ITS region was performed as described previously [22–24]. with minor modifications. Oligonucleotide primers BsppITS325s: 5′ CTTCAGATGATGATCCCAAGCCTTCTGGCG 3′ (Forward) and 543as: 5′ AATTGGTGGGCCTGGGAGGACTTG 3′ (Reverse) and probe BsppITS500probe: 5′ FAM-GTTAGAGCGCGCGCTTGATAAG -IABkFQ 3′ were utilized for these assays. Each 25 μl PCR reaction was prepared using 12.5 μl of SsoAdvanced™ Universal Probes Supermix (Bio-Rad, Hercules, CA), 0.2 μl of 100 μM of each forward primer, reverse primer, and TaqMan probe (IDT® DNA Technology, Coralville, IA), 7.5 μl of Ultra-Pure, molecular grade water (Genesee Scientific, San Diego, CA, USA), and 5 μl of DNA from each sample tested. Positive amplification was assessed by analysis of detectable fluorescence vs cycle threshold values.

Droplet digital PCR amplification of the *Bartonella* ITS region was conducted using the same primers and probes employed for qPCR, with minor modifications. The 22 μl final ddPCR reaction contained 11 μl of ddPCR™ Supermix for probes (no dUTP) (Bio-Rad, Hercules, CA), 0.2 μl each of 100 μM forward and reverse primers, 0.2 μl of 100 μM probe (IDT® DNA Technology, Coralville, IA), 3.8 μl of molecular grade water, 5 μl of DNA from each sample tested, and 1 μl of *Hin*dIII DNA restriction enzyme. Bio-Rad QuantaSoft Analysis Pro software was utilized to track and analyze the fluorescent drop distribution and positive detection threshold readings for each channel (FAM channel 1 for *Bartonella*, and HEX channel 2 for house-keeping gene amplification).

### Statistical analyses

Statistical analyses were performed in R (Version 4.1, R Core Team). For all categorical variables from the *Bartonella* spp. test results and self-reporting questionnaires, cases and controls were compared using a chi-squared test or a Fisher exact test (for small group numbers). For continuous variables, the groups were compared using a Wilcoxon rank-sum test for nonparametric data. For all statistical tests, the significance level was set to α = 0.05. Descriptive statistics were calculated for demographic, health and animal exposure history, and self-reported questionnaire data. The kappa statistic was used to assess agreement between serological and molecular testing modalities.

## Results

Over the course of a 2-year recruitment period, 70 controls and 11 cases were enrolled. After exclusions, 59 controls and 11 cases were used for data analysis. A total of 66 whole blood and 65 serum samples were obtained from controls, and a total of 11 whole blood and serum samples were obtained from cases. No samples were missing from cases, whereas five serum and four whole blood samples were missing from controls. The latter were due to difficulty in obtaining venous access. One additional serum sample was not tested due to excessive hemolysis following centrifugation which made the sample unusable for IFA. Six controls, including the participant with an excessively hemolyzed sample, were excluded from analyses due to scoring below 26 on the MoCA. One control was excluded after reporting use of psychiatric medication at the in-person session, and another control was not analyzed due to a lost sample.

Demographic and animal/insect exposure information for controls and cases are summarized in Table 1. Age, gender, work status, living situation, and residential environment did not differ significantly between cases and controls. The only characteristic that differed significantly between cases and controls was the highest level of education achieved (*p =* 0.014). Animal and insect exposures are also reported in Table 1. For both dogs and cats, there were no significant differences between cases and controls for current ownership, past ownership, or reports of bites/scratches. Additionally, there were no significant differences between cases and controls for reported exposure to potential insect vectors.

**Table 1:** Demographics and exposures.

	Control, *n* = 59	Case, *n* = 11	*p*
Demographics			
Age			0.668
*M* (SD)	68.6 (7.1)	67.6 (7.1)	
Sex			1
Male	18	3	
Female	41	8	
Race			0.210
White	52	9	
Black or African American	2	2	
Asian	4	0	
Other	1	0	
Work status			0.154
Not working by choice	44	7	
Seeking employment	1	0	
Working full-time	10	2	
Working part-time	3	0	
Unable to work due to health condition	0	1	
Other	1	1	
Highest education obtained			0.014*
Advanced degree	33	3	
Associate’s degree	1	1	
Bachelor’s degree	13	2	
Some college	5	1	
Some post undergraduate work	4	0	
High school diploma or equivalent	2	4	
Other	1	0	
Living situation			0.344
Lives alone	10	0	
Lives with partner/spouse/family/friends	49	11	
Living area			0.751
Suburban	35	7	
Urban	16	2	
Rural/Farm	2	0	
Rural/Wooded	6	2	
Reported Exposures			
Dog contact			
Currently own a dog	19	2	0.485
Has owned a dog	47	7	0.259
Bitten/scratched by dog	20	2	0.483
Cat contact			
Currently own a cat	14	3	1
Has owned a cat	39	6	0.505
Bitten/scratched by cat	32	5	0.745
Dog and cat Contact			
Currently own either	2	1	0.406
Has owned either	30	5	1
Bitten/scratched by either	14	2	1
No dog or cat contact			
Do not currently own either	28	7	0.513
Have never owned either	3	3	0.045*
Have never been bitten/scratched by either	21	6	0.315
Insect bites			
Flea	31	4	0.513
Biting fly	27	3	0.331
Mosquito	58	9	0.062
Spider	20	2	0.483
Mite	5	0	1
Bedbug	7	1	1
Lice	4	0	1
Bee	41	6	0.485
Wasp	37	5	0.328
Hornet	18	2	0.494

*p* values for comparison between cases and controls, using chi-squared tests or Fisher tests for proportions and Wilcoxon rank-sum test for continuous variables

*Statistically significant at *p* < 0.05.

Assessment results for cases and controls are summarized in Table 2. In agreement with expectations of the study design, MoCA scores for cases (*Mdn* = 22) compared to controls (*Mdn* = 28) were significantly lower, *W* = 645.5, *p* < 0.001, *r* = 0.63. Q-LES-Q-SF percentage scores for cases (*Mdn* = 71.4%) compared to controls (*Mdn* = 80.4%) were significantly lower, *W* = 508, *p* = 0.003, *r* = 0.355. Also, PHQ-9 scores for cases (*Mdn* = 4) compared to controls (*Mdn* = 1) were significantly higher, *W* = 174.5, *p* = 0.013, *r* = 0.30.

**Table 2:** Assessment results.

	Control, *n* = 59	Case, *n* = 11	*p*
MoCA *M* (*SD)*	27.93 (1.47)	21.34 (2.34)	< 0.001*
PHQ-9 *M* (*SD)*	1.70 (1.97)	5.00 (5.06)	0.013*
GAD-7 *M* (*SD)*	2.05 (2.25)	5.00 (5.51)	0.128
Epworth Sleepiness Scale, *M* (*SD)*	5.20 (2.92)	6.64 (4.06)	0.214
Q-LES-Q-SF *M* (SD)	80.81% (8.99%)	68.02% (15.56%)	0.003*

Reported mean, standard deviation, and *p* values for comparison between cases and controls, using Wilcoxon rank- sum test

*Statistically significant at *p* < 0.05.

MoCA, Montreal Cognitive Assessment; PHQ-9, Patient Health Questionnaire 9-item; GAD-7, Generalized Anxiety Disorder 7-item; Q-LES-Q-SF, Quality of Life Enjoyment and Satisfaction Questionnaire Short-Form

**Fig 1:**
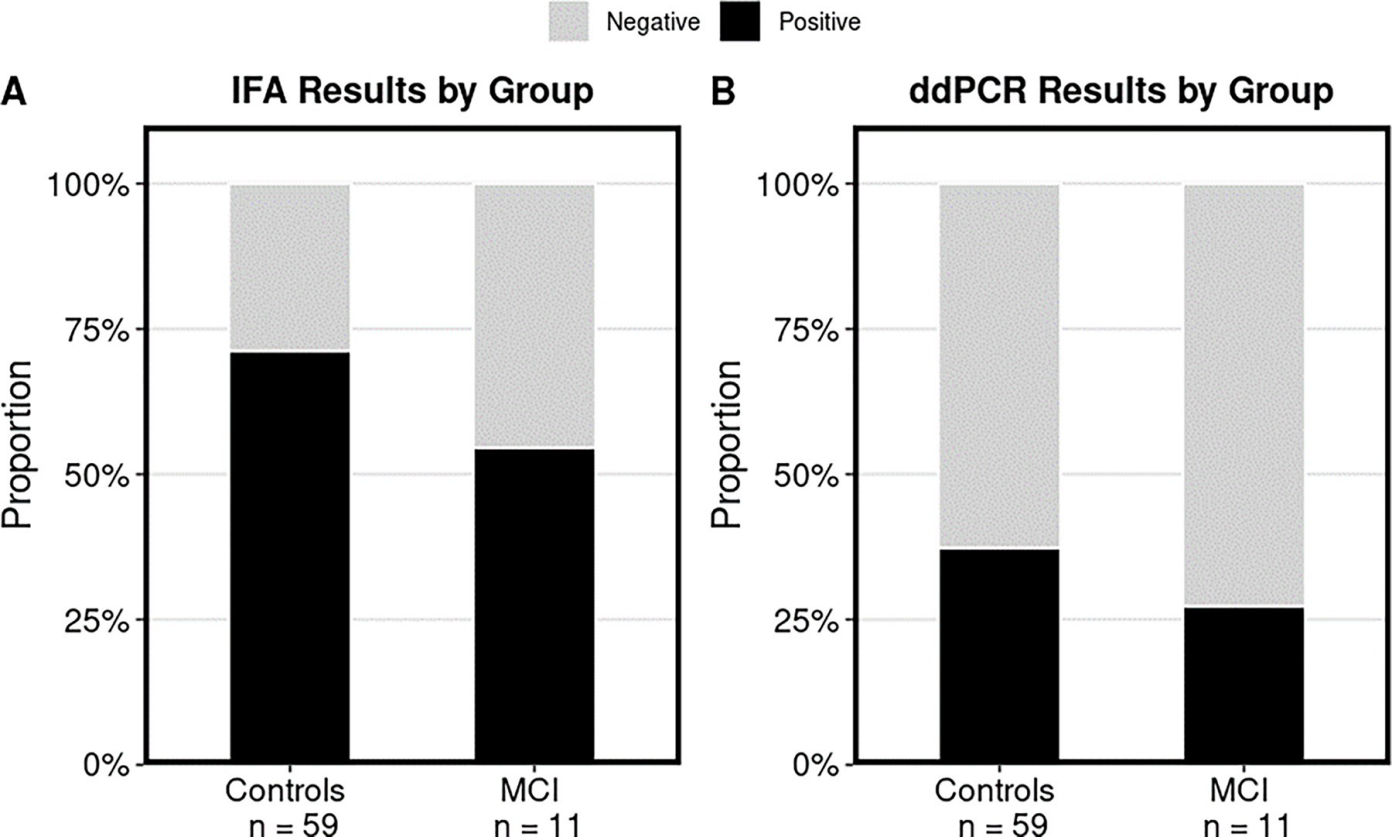
*Bartonella* spp. testing results. Panel A shows IFA results, panel B shows ddPCR results. Quantitative PCR results are not shown since no cases tested positive. *Black*, positive test result; *gray*, negative test result. Proportions are displayed for better visualization due to unequal sample sizes between groups.

For the primary aim, *Bartonella* spp. testing results are summarized in Fig 1 and Table 3. There was no significant difference between cases and controls for *Bartonella* spp. infection, based on both ddPCR and qPCR testing (*p* = 0.735 and *p* = 1, respectively). Three cases tested positive for active infection by ddPCR; however, qPCR failed to detect *Bartonella* spp. DNA from any case blood or 7-, 14-, and 21-day samples. When a sample was defined as PCR positive if either PCR modality generated a positive result, no significant difference was found between groups (*p* = 0.107). A surprisingly high level of active infection measured via ddPCR was found in controls, with 22 of 59 (37%) controls testing positive. Five controls tested positive for *Bartonella* spp. infection by qPCR (8.47%); however, only one of these aligned with a corresponding positive ddPCR result. Of the five positive controls by qPCR, *B*. *quintana* was the most common species (*n* = 4). The other species detected was *B*. *henselae*. Positive ddPCR results were confirmed in eight additional controls using species-specific probes. Two were infected with *B*. *quintana*, two with *B*. *henselae*, three with *B*. *alsatica*, and one control was co-infected with *B*. *henselae* and *B*. *vinsonii* subsp.

**Table 3:** *Bartonella* spp. PCR results.

	Control, *n* = 59	Case, *n* = 11
qPCR-positive	5 (0.08%)	0
ddPCR-positive	22 (37%)	3 (27%)
qPCR- & ddPCR-positive	1 (0.02%)	0

Number (and percentage) of participants in each group that tested positive for *Bartonella* spp. on qPCR, ddPCR, or both.

Based on IFA seroreactivity (≥ 1:64 titer), *Bartonella* spp. exposure was not significantly different between cases and controls: 71% of controls and 55% of cases were seroreactive to one or more of the five *Bartonella* spp. antigens (*p* = 0.461). A summary of IFA results is reported in Table 4. Again, a surprisingly high level of exposure was found in controls. Analyses were also completed separately for each species or subspecies and no significant differences were found between the IFA antigen groups. Based on IFA, *B*. *henselae* (San Antonio 2) seroreactivity was the most common among controls [59%], while this and *Bartonella vinsonii* subsp. *berkhoffii* genotype II were the most common among cases, with both strains at 45%. Comparisons between PCR and IFA results are reported in Table 5. Disagreement with IFA was significant for both ddPCR (kappa = -0.16) and qPCR (kappa = -0.19).

**Table 4:** *Bartonella* spp. indirect fluorescent antibody results.

	Control, *n* = 59	Case, *n* = 11
Bvb I	27 (46%)	4 (36%)
Bvb II	26 (44%)	5 (45%)
BhSA2	35 (59%)	5 (45%)
Bk	22 (37%)	4 (36%)
Bq	29 (49%)	3 (27%)

Number (and percentage) of participants in each group who were seroreactive against each *Bartonella* species antigen tested. Bvb I, *Bartonella vinsoni*i subsp. *berkhoffii* genotype I; Bvb II, *Bartonella vinsonii* subsp. *berkhoffii* genotype II; Bh, *Bartonella henselae* San Antonio 2; Bk, *Bartonella koehlerae*; Bq, *Bartonella quintana*

**Table 5:** PCR vs. IFA agreement testing.

	*All Participants*	*Kappa*
ddPCR/IFA	*n = 70*	-0.16
Positive/positive	14	
Positive/negative	11	
Negative/positive	34	
Negative/negative	11	
qPCR/IFA	*n = 70*	-0.19
Positive/positive	3	
Positive/negative	2	
Negative/positive	45	
Negative/negative	20	

Agreement between PCR and IFA results based on Cohen’s kappa score. Negative values

indicate significant disagreement.

The most common symptoms for cases and controls based on the health history questionnaire are reported in Table 6. Cases reported a significantly higher number of total symptoms (*Mdn* = 4) than controls (*Mdn* = 2), *W* = 182.5, *p* = 0.021, *r* = 0.28. Total reported symptoms when a PCR result was positive (*Mdn* = 2) did not differ significantly from when PCR-testing was negative (*Mdn* = 2), *W* = 629, *p* = 0.685, *r* = 0.05.

**Table 6:** Most common health symptoms.

Control, *n* = 59		Case, *n* = 11	
Joint pain	22	Difficulty remembering	10
Insomnia	16	Anxiety/Panic Attacks	5
Difficulty remembering	13	Depressed mood	4
Fatigue	10	Irritability/Rage/Aggression	4

Counts of the most reported symptoms on the health history questionnaire, by controls and cases.

## Discussion

In contradiction to our hypothesis, participants with MCI were not more likely than controls to be infected with *Bartonella* spp. based on both ddPCR and qPCR test results. Additionally, *Bartonella* spp. exposure was not significantly different between MCI patients and controls based on IFA seroreactivity. For both PCR and IFA, a surprisingly high proportion of controls tested positive (22 of 59 (37%) for ddPCR; 42 of 59 (71%) for IFA). IFA and ddPCR results showed significant disagreement, which in addition to other factors may be a result of *Bartonella* antibodies suppressing circulating bacteria below ddPCR detection levels [25].

The limited rate of positive findings from qPCR testing across both groups underscores established distinctions between the two PCR techniques used. While ddPCR exhibits heightened sensitivity, it lacks the specificity provided by qPCR, which can discern specific pathogen species through Sanger sequencing when adequate quantities of pathogen DNA are amplified. Additionally, ddPCR offers direct and independent quantification of DNA, which has been found to provide greater reproducibility compared to qPCR [26]. Use of ddPCR also reduces the effect of inhibitory substances by sequestering target DNA among individual droplets [27]. In this study, ddPCR findings were strengthened by using species-specific probes to confirm ddPCR-positive results in eight controls.

No significant differences in animal or insect exposure were found between cases and controls. A recent study found no evidence that the risk of MCI among those with normal baseline cognition differed between pet caretakers and non-caretakers [28]. The present study corroborates these findings as there was no association found between MCI and dog/cat ownership, previous exposure, or bites/scratches. Similar to the results of this study, *Bartonella henselae* and *Bartonella quintana* are the two most prevalent species infecting humans worldwide [12]. Interestingly, one ddPCR-positive control exhibited coinfection with *B*. *henselae* and *B*. *vinsonii* subsp, a coinfection previously found in a veterinarian and his daughter, both of which reported neurological symptoms [29]. Detection of *B*. *alsatica*, a *Bartonella* spp. for which rabbits are the primary reservoir host in three participants was an unexpected finding. Infection with *B*. *alsatica* has been reported in association with culture-negative endocarditis, granulomatous lymphadenitis and vascular graft rejection patients with exposures to wild rabbits [12]. A significant difference in self-reported symptoms was found between groups with cases reporting more symptoms on the health history questionnaire than controls. Additionally, the MCI group had significantly higher PHQ-9 and lower Q-LES-Q-SF scores, indicating more depressive symptoms and a lower quality of life, respectively. Both findings support known symptomatology associated with MCI [30]. No association was found between *Bartonella* spp. infection and total reported health symptoms, which may align with the lack of difference found in infection levels between groups.

Another factor to consider is that we only looked for *Bartonella* spp. infection within whole blood and serum samples. While *Bartonella* spp. typically colonize and proliferate within the vascular endothelium before infecting erythrocytes [31], other migratory cell types and lymphatic vessels likely aid in the transport of *Bartonella* spp. [32]. In addition, *Bartonella* spp. can also locally manifest within the skin and bone leading to various skin diseases [33]. *Bartonella* spp. have been found in the cerebral spinal fluid of people presenting with central nervous system infection caused by *Bartonella* spp. [17, 34, 35]. Therefore, extracting and analyzing the cerebral spinal fluid would have been another suitable method to specifically test for neurological infection with *Bartonella* spp.

A noteworthy aspect of this study was the unexpectedly high occurrence of *Bartonella* spp. infection and exposure among the control group. Previous studies in healthy populations have found a wide range of seroreactivity. A recent case-control study in North Carolina also found a higher than anticipated seroprevalence in the control group at 92% [19]. In this study, we found that exposures to common sources of *Bartonella* spp. transmission such as cat bites/scratches, fleas and other known or suspected vectors were relatively high in both groups, which could provide a possible explanation for the high seroprevalence. Another study in North Carolina, however, found a low seroprevalence of 3% in controls [21]. When looking at other geographic regions, variability still appears high. A study conducted in healthy adults in Korea, for example, found a seroprevalence of 15% for *Bartonella henselae*, [36] while a study in veterinary workers from central Spain found a seroprevalence of 83% across six antigens [37]. Among these studies, a combination of factors varied and likely contribute to such disparate findings, including geographic region, laboratory methodologies, the study or patient populations tested, as well as the number of *Bartonella* species and strain types used for IFA testing. Since the present study offers another population to consider, being a largely elderly population in North Carolina tested across all five antigens, it is important to consider the potential influence of immune senescence and comorbidities associated with older age on infection prevalence within both the control and case groups. Immune senescence, characterized by gradual deterioration of the immune system with age, can lead to increased susceptibility to infections and altered immune responses [38]. Immunocompromised individuals, such as the elderly population with underlying health conditions, may exhibit higher bacteremia rates [39] which could facilitate the detection of *Bartonella* spp. through molecular techniques like PCR or culture. Additionally, in IFA detection of *Bartonella* spp., the potential for cross-reactivity with other bacterial organisms has been previously reported, which may have contributed to the high seroprevalence; however, such reports are not consistent and may be due to exposure to multiple pathogens or multiple *Bartonella* species [40, 41]. It is also important to consider that the *Bartonella* spp. bacterium is more prevalent than previously suspected, as evidenced by a study testing healthy blood donors in Brazil, which found Bartonella spp. DNA amplified from nearly 20% of asymptomatic donors in São Paulo when multiple bacterial genes were targeted by qPCR [42]. As testing modalities have become more sensitive, occult Bartonella bacteremia has been found in both healthy and sick individuals, but establishing the role of these organisms in disease causation will require a multifaceted approach. Future research could continue exploring the prevalence of *Bartonella* spp. in both the elderly and general population, as well as possible sources of variability.

The cases and controls in this study were recruited from the same geographic region with limited significant differences in other demographic factors, barring educational status, suggesting that most potential confounds were controlled for. However, there are several limitations to the interpretation of results that are important to consider. First, interpretability of our data is reduced by lack of statistical power due to the small sample size of 11 for the MCI group and the unequal sample sizes between groups. A larger sample size in future studies may be better able to detect potential differences in *Bartonella* spp. infection and exposure between groups. As described, low qPCR sensitivity is also a concern, as evidenced by the low number of participants that were qPCR-positive relative to ddPCR. Future studies could employ paired testing for all modalities to provide a better indication of detection accuracy. This study does not provide definitive evidence regarding the lack of a connection between *Bartonella spp*. and MCI, and future research could address the limitations described or explore *Bartonella* spp. infection in other neuropsychiatric disorders that have been associated with neuroinflammation. Given the high overall prevalence of *Bartonella* spp. found in this study and several others, as well as the evidence for long-standing infection in humans, [22] it is important to explore the potential role of these bacteria in CNS pathogenesis beyond MCI.

## Supporting information

S1 File Raw serology, PCR, and questionnaire data. This spreadsheet contains the serology, PCR, and questionnaire data collected for this study. Some subjects in the spreadsheet were not included in analysis for reasons described in the manuscript.
(CSV)

S1 Data (CSV)
